# Retraction: Ensemble learning for multi-class COVID-19 detection from big data

**DOI:** 10.1371/journal.pone.0321416

**Published:** 2025-04-07

**Authors:** The PLOS One Editors

The *PLOS One* Editors retract this article [1] due to concerns about peer review integrity, authorship, and adherence to the journal’s publication requirements on reporting and data availability. We regret that the issues were not addressed prior to the article’s publication.

SK, AS, MUT, and MB did not agree with the retraction. BQ either did not respond directly or could not be reached.
